# Delayed Diagnosis of Respiratory Syncytial Virus Infections in Hospitalized Adults: Individual Patient Data, Record Review Analysis and Physician Survey in the United States

**DOI:** 10.1093/infdis/jiz236

**Published:** 2019-05-09

**Authors:** Nelson Lee, Edward E Walsh, Ian Sander, Robert Stolper, Jessica Zakar, Veronique Wyffels, David Myers, Roman Fleischhackl

**Affiliations:** 1 Division of Infectious Diseases, Department of Medicine, University of Alberta, Edmonton, Canada; 2 Infectious Disease Division, Department of Medicine, University of Rochester School of Medicine and Dentistry, New York; 3 IQVIA Consulting Services, Cambridge, Massachusetts; 4 Janssen Pharmaceutica, Beerse, Belgium; 5 Janssen-Cilag, Solna, Sweden; 6 Medical Department, Janssen-Cilag, Vienna, Austria

**Keywords:** respiratory syncytial virus, record review, adult, diagnosis, acute respiratory infection

## Abstract

**Background:**

Despite the prevalence of respiratory syncytial virus (RSV) in adults hospitalized with acute respiratory infections, guidelines for the diagnosis and management of RSV have not been established. This analysis evaluated the role and timeliness of RSV diagnostic testing and its potential impact on clinical outcomes.

**Methods:**

We analyzed individual patient data from hospitalized adults with confirmed RSV infections during 2 North American RSV seasons. Participating physicians reported clinical, virologic diagnosis, and outcome variables using a standardized online case form.

**Results:**

Across 32 US states, 132 physicians reported 379 RSV cases. Polymerase chain reaction–based diagnostics were the most common type of test ordered (94.2%) with <5% ordered specifically to diagnose RSV. Most tests (67.6%) were ordered in hospital wards or intensive care units. Overall, 47.4%, 30.9%, and 21.7% of patients had RSV diagnosed <12, 12‒24, and >24 hours after hospital admission, respectively. Later diagnosis was associated with longer hospital stays (n = 145; *R* = +0.191; *P* < .05) and greater antibiotic use.

**Conclusion:**

Diagnosis of RSV infection in hospitalized adults is often delayed, which may affect clinical management and outcomes. Our findings indicate the need to improve the diagnostic strategies in this patient population.

## Teaser

Diagnosis of respiratory syncytial virus infection in hospitalized adults is often delayed, which may affect clinical management and outcomes such as hospital stays and antibiotic use. Our findings indicate the need to improve the diagnostic strategies in this patient population.

Acute respiratory infections caused by respiratory syncytial virus (RSV) are common in adults, causing excessive hospitalizations and deaths worldwide [1]; the estimated disease burden is similar to that of seasonal influenza [2]. Besides debilitating, protracted symptoms, RSV infections can result in respiratory failure, prolonged hospitalization, and high mortality rates, similar to outcomes in influenza [2–16]. In older adults, 86–254 hospitalizations per 100 000 persons are attributable to RSV [2‒4, 6–13, 15, 16]. Patients with immunocompromising conditions [5, 14, 17–19] and those with underlying cardiopulmonary disease [11, 20] are at particularly high risk for severe diseases and complications.

Despite growing knowledge on its disease burden, RSV infections in adults are frequently underrecognized in clinical settings [3, 5]. Because of low clinical suspicion, tests that specifically target RSV may not be requested or substantially delayed; unlike for pediatric patients, no guideline for the diagnosis and management of RSV in adults is available [3]. Furthermore, some accessible RSV tests may lack sensitivity (eg, rapid antigen tests) [21, 22] or have long turnaround times that limit their utility in acute care units (eg, emergency departments [EDs]) [23]. Emerging data suggest that prompt, polymerase chain reaction (PCR)–based diagnosis of viral acute respiratory infections in the ED or in the hospital may allow optimization of clinical management and impact on patient outcomes [24‒27]. Together with the advances in antiviral development [28], it is important to clarify the barriers to RSV diagnosis and their potential effects on patient care.

In the current analysis, we aimed to describe the clinical characteristics, presenting symptoms, and the methods and timing of virologic diagnosis in adults hospitalized for RSV infections, using data from record review and a physician survey across the United States. The associations of these parameters with management decisions and the hospital course were analyzed. Such results may provide useful insights and allow optimization of RSV diagnostic strategies in this patient population.

## METHODS

### Design

We conducted a retrospective, individual patient data, record review analysis on the diagnosis of RSV infections in an adult subpopulation (hospitalized patients) in the United States. Deidentified patient case data from 2 North American RSV seasons (1 October 2014 to 21 October 2016) were entered by the treating hospital-based physician into a standardized online case form. Each physician entered data for between 1 and 3 patients who had a confirmed diagnosis of RSV. The case form was customized to capture available details standard in medical records, such as length of stay (LOS), timing of tests, and medical treatments, together with clinical information specific to RSV disease (eg, presenting symptoms) (see Supplementary Materials for individual variables). The analysis was conducted in accordance with the guidelines set out by the US Health Insurance Portability and Accountability Act (1996), and was exempt from formal review by the New England Independent Review Board. Owing to the study’s retrospective nature, and because the data contained no identifying characteristics, informed consent was not requested or required from patients whose data were used.

### Inclusion Criteria for Respondents

**Figure 1.**
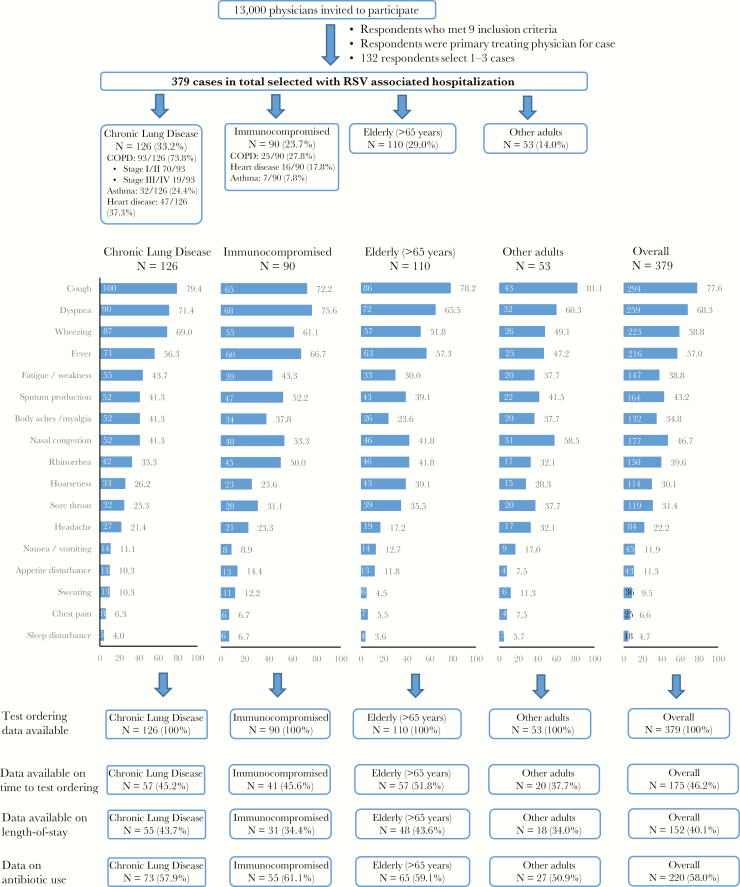
Flow of patients with respiratory syncytial virus, risk group classification, and presenting symptoms at time of hospital admission. Abbreviation: COPD, chronic obstructive pulmonary disease.

Approximately 13 000 physicians were invited through market research panels to participate in the analysis (Figure 1). There was no follow-up of physicians who did not respond to the invitation. Physicians were provided with a small honorarium ($135–$200, depending on specialty) as an incentive to complete the survey. Respondents were required to meet a total of 9 inclusion criteria in order to participate in the analysis (see Supplementary Materials).

### Random Selection and Inclusion Criteria for Cases

Eligible respondents were asked to select up to 3 patient cases for whom they were the primary treating physician. To prevent physicians from selecting the “most memorable,” “most typical,” “most rare,” or “most recent” cases, respondents were allocated a random letter of the alphabet for each patient and asked to begin searching their patient files in each instance for eligible patients whose last name began with the given letter. To be eligible, patients were required have a positive test result for RSV within the past 2 seasons, to be aged ≥18 years, and not to be currently participating in a clinical trial. For reporting purpose, cases were classified into 1 of 4 mutually exclusive “risk groups,” as follows: chronic lung diseases, immunocompromised, elderly (>65 years and without immunocompromising conditions and chronic lung diseases), and “other adults” (younger patients without the above conditions) (see Supplementary Materials).

### Data Analyses

Descriptive results of baseline case demographics, underlying conditions, presenting symptoms, diagnostic tests used, site of test ordering, time intervals for testing and reporting, hospital LOS, and antibiotic use, where available, are reported for all cases, and according to the 4 risk groups. Percentages or means with standard deviations (SDs) were reported, whenever appropriate. The overall time interval to diagnosis (“admission-to-result” interval), and the “admission-to-test-ordering” and “test-ordering-to-result” intervals were analyzed (see Supplementary Materials for details). Simple least-squares linear regression analysis was used to examine correlations between LOS and time to diagnosis. Pearson correlation coefficients (*R*) were calculated based on variance from the best-fit linear regression. Comparisons between diagnosis-related time intervals across risk groups were performed using 1-way analysis of variance (ANOVA); their (categorical) associations with LOS and duration of antibiotics use were examined using paired *t* tests. Statistical analyses were performed using IBM SPSS Statistics software (version 23).

## RESULTS

### Respondent Physicians’ Demographics

More than 13 000 physicians were invited to participate; approximately 7% responded and completed screening. In total, 132 physicians across 32 states met the criteria and actively participated in the survey (Supplementary Table 1). All responding physicians (mean [SD] age, 45 [7.8] years; 104 [78.8%] male) worked in a hospital setting, practicing predominantly in urban (n = 76 [58.0%]) or suburban (n = 53 [40.6%]) settings, and working mostly in teaching hospitals (n = 82 [62.1%]). The primary medical specialties reported were pulmonologist (n = 34 [25.8%]), infectious disease specialist (n = 32 [23.9%]), oncologist (n = 25 [18.9%]), intensivist (n = 17 [12.9%]), hospitalist (n = 16 [12.1%]), and internal medicine or general practitioner (n = 8 [6.1%]). The mean (SD) number of beds in the respondents’ primary hospital was 525 (408). Only 12 hospital sites (9.1%) were reported to have an established RSV testing protocol.

### Patient Case Demographics and Presenting Features

A total of 379 eligible patient cases were submitted, with 213 (56.2%) coming from physicians in an integrated delivery network. Patients were reported according to 4 risk groups based on their underlying characteristics: chronic lung diseases (n = 126 [33.2%]), immunocompromising conditions (n = 90 [23.7%]), elderly (aged >65 years; the majority had cardiovascular diseases) (n = 110 [29.0%]), and other adults (n = 53 [14.0%]) (Table 1 and Figure 1; patient comorbid conditions are detailed in the Supplementary Materials). Among the subset with chronic lung diseases, 93 of 126 patients (73.8%) had chronic obstructive pulmonary disease (COPD; stage I/II, n = 70; 75.3%; stage III/IV, n = 19; 21.2%; Global Initiative for COPD classification) (https://goldcopd.org/wp-content/uploads/2018/02/WMS-GOLD-2018-Feb-Final-to-print-v2.pdf), 47 (37.3%) had concomitant heart diseases, and 32 (25.4%) had asthma.

**Table 1.** Patient Demographic and Clinical Characteristics by Risk Group (N = 379)

Characteristic	Patients, No. (%)^a^				
	Chronic Lung Diseases (n = 126)	Immunocompromised (n = 90)	Elderly (n = 110)	Other Adults (n = 53)	Overall (N = 379)
Age, mean (SD; range) y	63 (15; 18–95)	57 (15; 19–82)	70 (5; 65–88)	41 (12; 20–64)	60 (16; 18–95)
Male sex	68 (54)	51 (57)	60 (55)	32 (60)	211 (55.6)
Race/ethnicity					
White	71 (56.3)	54 (60.0)	66 (60.0)	29 (54.7)	220 (58.0)
Black/African American	34 (26.9)	20 (22.2)	22 (20.0)	16 (30.2)	92 (24.3)
Hispanic/Latino	12 (9.5)	10 (11.1)	10 (9.1)	3 (5.7)	35 (9.2)
Native American or Alaska Native	3 (2.4)	1 (1.1)	2 (1.8)	2 (3.8)	8 (2.1)
Asian	3 (2.4)	2 (2.2)	9 (8.2)	3 (5.7)	17 (4.5)
Native Hawaiian or other Pacific Islander	0	1 (1.1)	1 (0.9)	0	2 (<1)
Don’t know	3 (2.4)	3 (3.3)	0	0	6 (1.6)
Smoking status					
Current smoker	47 (37.3)	19 (21.1)	19 (17.3)	27 (50.9)	112 (29.6)
Previous smoker	60 (47.6)	38 (42.2)	59 (53.6)	14 (26.4)	171 (45.1)
Never smoked	16 (12.7)	30 (33.3)	28 (25.5)	10 (18.9)	84 (22.2)
Don’t know	3 (2.4)	3 (3.3)	4 (3.6)	2 (3.8)	12 (3.2)
Caregiver requirements before hospitalization					
Did not need caregiver	66 (52.3)	53 (58.8)	59 (53.6)	38 (71.6)	216 (57.0)
Relied on spouse/partner	42 (33.3)	24 (26.7)	33 (30.0)	11 (20.8)	110 (29.0)
Relied on child	9 (7.1)	6 (6.7)	8 (7.2)	4 (7.5)	27 (7.1)
Relied on professional caregiver/in-home nurse	9 (7.1)	7 (7.8)	10 (9.1)	0 (0)	26 (6.7)

Abbreviation: SD, standard deviation.

^a^Data repesent no. (%) of patients unless otherwise specified.

Patients’ presenting symptoms at the time of hospital admission are described in Figure 1. Cough (n = 294 [77.6%]), dyspnea (n = 259 [68.3%]), and wheezing (n = 223 [58.8%]) were the most frequently reported symptoms. Fever was seen in a higher proportion of patients in the immunocompromised group (n = 60 [66.7%]) than in the other 3 groups (n = 156 [47.1%‒57.3%]; *P* = .03). The overall incidence of symptoms typical of upper respiratory tract infection was low (rhinorrhea, n = 148 [39.1%]; sore throat, n = 119 [31.4%]).

### Diagnostic Procedures and Tests

Although 277 of 379 patients (73.1%) were admitted via the ED, only 112 of 379 patients (29.6%) had viral diagnostic tests ordered in the ED; most tests (67.6%) were ordered later, either in the hospital wards (n = 189 [49.9%]) or in the intensive care unit (ICU) (n = 67 [17.7%]) (Table 2). In hospital sites with an established RSV testing protocol (12 sites, 31 patients), a higher proportion of patients were tested in the ED (51.6% vs 39.8% in the 246 patients without an established protocol; *P* = .04).

**Table 2.** Diagnostic Procedures and Tests for Respiratory Syncytial Virus Infection Used in Hospitalized Patients

Diagnostic Procedure or Test	Patients, No. (%)				
	Chronic Lung Diseases (n = 126)	Immunocompromised (n = 90)	Elderly (n = 110)	Other Adults (n = 53)	Overall (N = 379)
RSV test order location					
In the ED	45 (35.7)	20 (22.2)	34 (30.9)	13 (24.5)	112 (29.6)
In the hospital ward	57 (45.2)	48 (53.3)	56 (50.9)	28 (52.8)	189 (49.9)
In the ICU	19 (15.1)	19 (21.1)	20 (18.2)	9 (17.0)	67 (17.7)
Others	0	1 (1.1)	0	0	1 (<1)
Don’t know	5 (4.0)	2 (2.2)	0	3 (5.6)	10 (2.6)
RSV test ordered specifically?					
Yes	3 (2.4)	5 (6.6)	6 (5.4)	3 (5.6)	17 (4.5)
No	98 (77.8)	68 (75.6)	88 (80.0)	43 (81.1)	297 (78.3)
Others	0	0	1 (0.9)	0	1 (<1)
Don’t know	25 (19.8)	17 (18.9)	15 (13.6)	7 (13.2)	64 (16.7)
No. of diagnosis methods used					
Don’t know	15 (11.9)	5 (6.6)	2 (1.8)	3 (5.6)	25 (6.6)
1	95 (75.4)	80 (88.9)	101 (91.8)	48 (90.6)	324 (85.5)
2	9 (7.1)	4 (4.4)	6 (5.5)	2 (3.8)	21 (5.5)
3	4 (3.2)	0	1 (0.9)	0	5 (1.3)
4	3 (2.4)	1 (1.1)	0	0	4 (1.1)
Diagnostic method^a^					
Respiratory virus panel including RSV (multiplex PCR)	88 (69.8)	67 (74.4)	77 (70.0)	37 (70.0)	269 (71.0)
Specific RSV PCR assay	20 (15.9)	17 (18.9)	21 (19.1)	8 (15.1)	66 (17.4)
Rapid antigen detection RSV assay	13 (10.3)	4 (4.4)	9 (8.1)	3 (5.6)	29 (7.7)
Central laboratory PCR RSV assay	10 (7.9)	3 (3.3)	6 (5.5)	4 (7.5)	23 (6.1)
Cell culture detection RSV assay	4 (3.2)	0	3 (2.7)	0	7 (1.8)
Immunofluorescent RSV assay	2 (1.6)	1 (1.1)	0	0	3 (<1)
Don’t know	15 (11.9)	5 (6.6)	2 (1.8)	3 (5.6)	26 (6.9)
Sampling method					
Nasopharyngeal swab	72 (57.1)	57 (63.3)	71 (64.5)	25 (47.1)	225 (59.4)
Nasal swab	38 (30.2)	21 (23.3)	23 (20.9)	18 (34.0)	100 (26.4)
Sputum	14 (11.1)	4 (4.4)	12 (10.9)	7 (13.2)	37 (9.7)
Throat swab	9 (7.1)	1 (1.1)	13 (11.8)	5 (9.4)	28 (7.4)
Bronchoalveolar lavage	7 (5.6)	11 (12.2)	5 (4.5)	3 (5.6)	26 (6.8)
Nasal wash	4 (3.2)	4 (4.4)	7 (6.3)	3 (5.6)	18 (4.7)
Middle turbinate swab	2 (1.6)	0	2 (1.8)	1 (1.9)	5 (1.3)
Don’t know	7 (5.6)	3 (3.3)	1 (0.9)	2 (3.8)	13 (3.4)

Abbreviations: ED, emergency department; ICU, intensive care unit; PCR, polymerase chain reaction; RSV, respiratory syncytial virus.

^a^Percentages may exceed 100% because >1 diagnostic test may have been used in some patients.

Nearly all patients (94.2%) had their diagnosis of RSV confirmed at least in part via PCR-based diagnostic methods (Table 2), including multiplex PCR respiratory virus panels (269 of 379 patients [71.0%]), rapid PCR assays (66 of 379 [17.4%]), and central laboratory PCR assays (23 of 379 [6.1%]). Rapid antigen detection assays were used in <7.7% (29 of 379 patients). Nearly all were combination tests in which RSV was one of the viral pathogens that could be detected. In most cases, the diagnosis of RSV was based on a single test method (324 of 379 [85.5%]), but in 30 patients, ≥2 tests were used for diagnosis (30 of 379 [7.9%]), and this was similar across all risk groups (Table 2). Nasopharyngeal (n = 225 [59.4%]) or nasal (n = 100 [26.4%]) swab samples were the samples used most frequently for RSV testing. Lower respiratory tract samples, including sputum and bronchoalveolar lavage samples, were used in 9.8% and 6.9% of patients, respectively.

In the great majority of cases, the treating physicians reported that the tests were ordered as an investigation for viral causes of respiratory infections (n = 297 [78.3%]), and only 17 (4.5%) were specifically ordered with the primary intention to diagnose RSV infection.

### Variability in Testing Times

#### Interval Between Hospital Admission and Test Ordering

**Figure 2.**
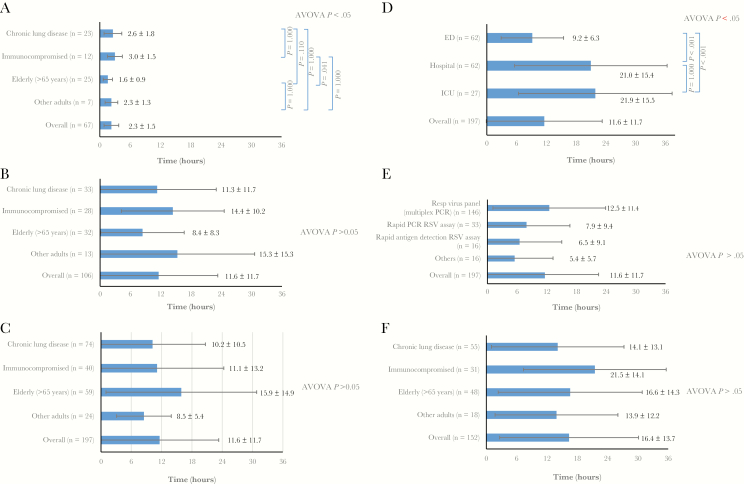
*A, B,* Time intervals between hospital admission and test ordering for tests requested in the emergency departments (ED) (*A*) or in the hospital ward or intensive care unit (ICU) (*B*), shown according to the 4 risk groups. *C‒E,* Intervals between test ordering (in hospital wards or ICU) and receipt of respiratory syncytial virus (RSV) result, shown according to the 4 risk groups (*C*), location of test request (*D*), and diagnostic methods (*E*). *F,* Intervals between admission and receipt of RSV result, shown according to the 4 risk groups. Data are shown as mean values with standard deviations. Abbreviations: ANOVA, analysis of variance; PCR, polymerase chain reaction.

The time of viral test ordering was recorded in 175 patients (46.2%); of these, 67 (38.2%) had a test ordered in the ED, and 75 (42.9%) and 33 (18.9%) had a test ordered on the hospital ward or in the ICU, respectively. If ordered in the ED, the mean (SD) time interval between admission and test ordering was 2.3 (1.5) hours (Figure 2A). In subanalyses, elderly patients seemed to have shorter delay in test ordering in the ED (n = 25; mean [SD] time, 1.6 [0.9] hours), compared with other risk groups (ANOVA; *P* = .03). If testing was ordered in the wards or ICU (n = 106), the interval was significantly longer (mean [SD], 11.6 [11.7] hours) than in the ED (*P* = .01) (Figure 2B). The latter included patients transferred from the ED (n = 56; mean [SD] interval, 17.4 [13.6] hours) and those directly admitted (n = 50; 5.9 [6.8] hours). There was no statistically significant difference in delay across risk groups when the tests were ordered in wards or the ICU (Figure 2B).

#### Interval Between Test Ordering and Receipt of Result

The mean (SD) time interval between test ordering and receipt of result was 11.6 (11.7) hours (n = 197) (Figure 2C). No statistically significant difference was found across the 4 risk groups. The interval was significantly shorter for tests ordered in the ED (mean [SD], 9.2 [6.3] hours) than for those ordered in either the ICU (21.9 [15.5] hours; *P *< .001) or the hospital (21.0 [15.4] hours; *P *< .001) (Figure 2D). There was a trend showing that the interval to receipt of result may be longest with the multiplex PCR respiratory viral panel (n = 146; mean [SD] interval, 12.5 [11.4] hours) compared with other test methods (mean, 5.4‒7.9 hours), however statistical significance was not reached (ANOVA; *P* > .05) (Figure 2E).

#### Interval Between Hospital Admission and Receipt of Result

The mean (SD) time interval from admission to receipt of result was 16.4 (13.7) hours. Across the 4 risk groups, there was considerable variability, with no statistically significant difference found (n = 152) (Figure 2F).

Overall, 47.4%, 30.9%, and 21.7% of patients received the diagnosis <12, 12–24, and >24 hours after hospital admission, respectively. The proportions of infections diagnosed in <12 hours were 43.3%, 70.3%, and 84.6% with multiplex PCR respiratory viral panel, RSV PCR assay, and rapid antigen tests, respectively (Supplementary Materials).

### Time of RSV Diagnosis and Hospital Course

#### LOS in Hospital

Overall, the mean (SD) LOS was 7.2 (5.3) days (N = 376). There were no significant differences between risk groups, with a mean (SD) LOS of 7.6 (5.8), 7.4 (5.2), 7.1 (5.7), and (6.4 (5.5) in the chronic lung disease, immunocompromised, elderly, and “other adults” groups, respectively.

**Figure 3.**
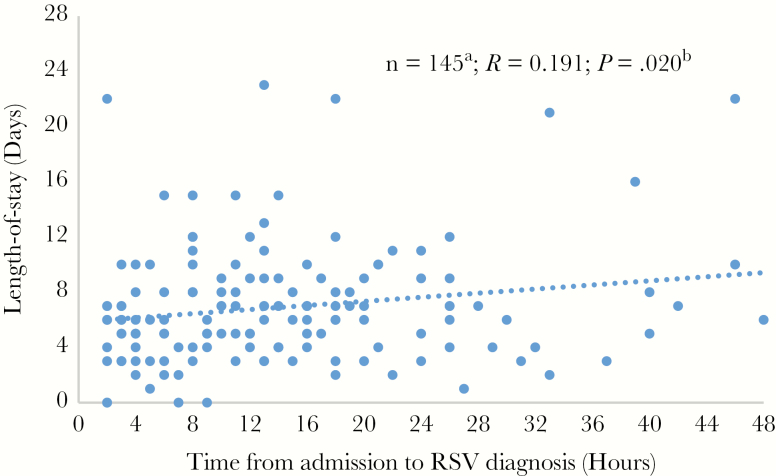
Regression analysis of length of stay (LOS) in hospitals and time to respiratory syncytial virus (RSV) diagnosis. The interval from admission to RSV diagnosis was reported by only 145 respondents. In addition, patients tested >4 days after admission (because diagnosis after more than half the typical LOS is unlikely to affect LOS) and those who stayed in the hospital for >3 weeks (threshold set 3 standard deviations above the median LOS) were excluded from the analysis (n = 7). Pearson correlation coefficients (*R*) were calculated based on variance from the best-fit linear regression (simple least-squares linear regression in IBM SPSS Statistics software, version 23). The *P* value for the relationship between hospital LOS and interval to diagnosis was calculated using 1-way analysis of variance.

**Figure 4.**
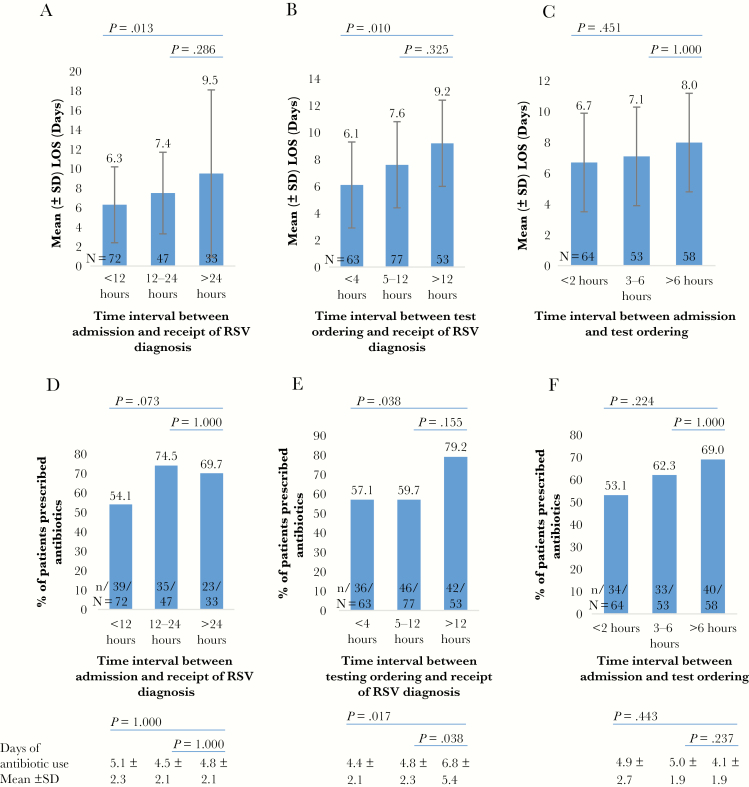
*A–C,* Relationships between length of hospital stay (LOS) and time intervals from admission (*A*) and test ordering (*B*) to receipt of respiratory syncytial virus (RSV) diagnosis and from admission to test ordering (*C*). *D–F,* Relationships between rate of antibiotic use and intervals from admission (*D*) and test ordering (*E*) to diagnosis and from admission to test ordering (*F*). Durations of antibiotic treatment are shown at the bottom of the panel. (See Supplementary Materials for detailed description of these time intervals). Abbreviation: SD, standard deviation.

Data for the LOS and time of RSV diagnosis (admission-to-result interval) were reported in 152 of 379 patients (40.1%). Regression analysis showed a significant correlation between time to RSV diagnosis and LOS (*R* = +0.191; *P* = .02) (Figure 3). The shortest mean (SD) LOS was observed among patients with infection diagnosed within <12 hours (6.3 [3.9] days), compared with those with diagnoses after >24 hours (9.5 [8.6] days; *P* = .01) or 12–24 hours (7.4 [4.2] days; *P* = .29) after admission (Figure 4A). Subanalyses revealed an association between LOS and the interval from test ordering to result, with the shortest LOS observed in those receiving a diagnosis within <4 hours (*P* = .01) (Figure 4B). Moreover, a trend toward shorter LOS was observed in patients whose tests were ordered early, within 2 hours after admission (Figure 4C). Patients tested in the ICU had significantly longer LOS (mean [SD], 9.4 [7.0] days) than those tested in the hospital wards or the ED (6.8 [5.3] days; *P* < .05).

#### Antibiotic Use and Duration

**Figure 5.**
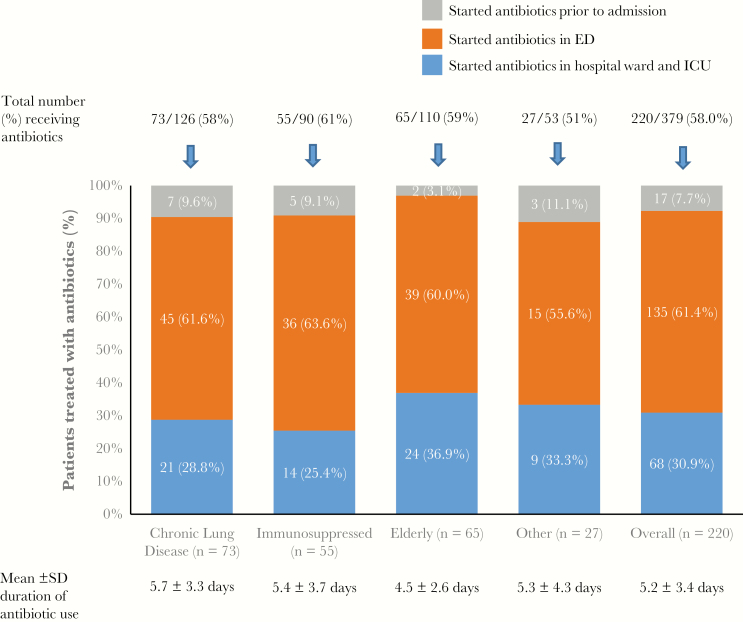
Rate and duration of antibiotic use in hospitalized patients with respiratory syncytial virus, shown according to the 4 risk groups. Abbreviations: ED, emergency department; ICU, intensive care unit; SD, standard deviation.

Overall, 220 of 379 patients (58.0%) had received antibiotic treatment, starting in the ED for the majority (Figure 5). The mean (SD) duration of antibiotic use was 5.2 (3.4) days, and this was similar across the risk groups. We found a higher rate of antibiotic use (79.2% vs 57.1%; *P* = .04) and a longer duration of treatment (mean [SD], 6.8 [5.4] vs 4.4 [2.1] days; *P* = .02) in patients with a delay of >12 hours before diagnosis (ie, test-ordering-to-result interval) than in those whose infection was diagnosed within <4 hours. Similar trends of association were observed for the admission-to-result and admission-to-test-ordering intervals (Figure 4D–4F and footnotes). Among the 187 of 220 patients who received antibiotics in the hospital, only 24.6% (46 of 187) had culture-confirmed bacterial infection, with clinically suspected infection in 47.1% (88 of 187). The remainder (28.3%) were reported to have no bacterial infection or to have a clinically uncertain status.

## DISCUSSION

In the current analysis, we found that RSV infections in hospitalized adults are frequently diagnosed late in the illness, despite the availability of rapid molecular tests. The missed opportunities to recognize the infection early in the hospital course may influence clinical decision making and negatively affect patient outcomes. Our findings indicate the need to establish efficient, pragmatic diagnostic strategies for RSV diseases in this patient population.

Our results showed that almost two-thirds of patients had RSV infection diagnosed only after admission to the hospital ward or ICU, and in more than half of the cases, the diagnosis was made >12 hours after admission, with one-fifth of diagnoses delayed for ≥24 hours. A combination of factors, including low clinical suspicion and the times required for sample collection and transport, test turnaround, and reporting can all contribute to such delay. The clinical features of RSV infection in adults are indistinguishable from other viral (and bacterial) causes of acute respiratory infection, so virologic tests are necessary to establish the diagnosis [11, 12]. Moreover, most patients do not present with typical upper respiratory tract symptoms (eg, rhinorrhea, sore throat), and fever can be absent [12]. In approximately 7%‒10% of cases, lower respiratory tract samples were submitted for diagnosis as disease progressed. Clinicians’ perception, practice, and diagnostic approaches for RSV infections remain heterogeneous across hospitals in the United States, and likewise elsewhere [11, 12]. Our findings underscore such challenges; notably, in a minority of sites where a diagnostic protocol is implemented, more infections were diagnosed at the earliest opportunity while patients were receiving care at the ED.

We found that PCR-based tests were the most widely used diagnostic method for RSV (used in 94% of cases). This probably reflects the increased accessibility to these molecular assays and the fact that physicians are more aware of their superior accuracy compared with antigen tests. Recently, the US Food and Drug Administration has reclassified rapid influenza virus antigen detection test systems from class I to class II devices, owing to their poor diagnostic performance and low sensitivity [29]. RSV antigen tests are also known to have varying and generally low sensitivities [30]. Most physicians reported that the primary intention of ordering tests was not specifically for RSV but to diagnose a viral causes for respiratory infections, which is consistent with the frequent ordering of multiplex PCR assays (70%). Notably, the use of PCR assays did not seem to be associated with the shortest turn-around-time, with nearly 60% of patients receiving the diagnosis >12 hours after admission. This probably reflects the logistical challenge in many hospitals, where assays are performed in batches in central laboratories only once, or at most twice, a day.

In addition, our results showed that early RSV detection was weakly associated with shorter hospital LOS, as well as a reduced rate and duration of antibiotic use, which warrants confirmation in prospective studies. These findings add to the growing evidence that prompt diagnosis of a viral cause may better inform clinical decision making and lead to improved patient outcomes, including reductions in hospital admissions, ED triage time, duration of isolation, total LOS, ancillary laboratory tests and imaging, and antimicrobial use [31‒34]. A randomized controlled trial reported fewer in-hospital antibiotic prescriptions or their earlier termination with rapid virologic diagnosis [34]. Our findings are consistent, and further indicate the feasibility of such an approach among patients with RSV infection, given that majority of prescriptions were considered empirical by the physicians. In most studies, the newer, point-of-care rapid molecular tests are used, either at the ED or in the hospital, as reviewed elsewhere [1]. Several of these tests have already received the Food and Drug Administration Clinical Laboratory Improvement Amendments of 1988 waiver for RSV and influenza/RSV detection [30]. Evaluation of their clinical utility is ongoing. At present, there is no established therapy for RSV; however, if available [21, 35], prompt diagnosis of RSV infection (and discrimination from influenza) could guide specific antiviral intervention [34]. Given these potential benefits, we propose the study of new diagnostic strategies that are efficient, pragmatic, and cost-effective in hospitalized patients with RSV infection.

Our analysis provides useful insights into the characteristics and virologic testing in adult patients with RSV across hospitals in the United States during 2 consecutive seasons. Patient “record-based” research is a valuable method for collecting rich, geographically diverse, real-world data from responding physicians’ practices. The customized case form enabled the capture of details standard in medical records (eg, LOS, tests used, and treatment) and additional clinical and laboratory parameters (eg, test turn-around time). Results of supportive therapies and the course of illness will be reported in a separate analysis. Our analysis is limited by its retrospective nature and the fact that more than half of respondents were from teaching hospitals; however, case selection and recall biases were minimized by our current design (see Methods). Furthermore, data on hospital LOS and the timing of diagnosis were available in only a subset of patient records. Patient characteristics (ie, risk group) did not seem to significantly affect LOS; however, it would be difficult to control for other potential confounders, such as disease severity, or to determine casual relationships with test timing in this observational study. Further studies with prospective, randomized controlled design are indicated [34].

 In conclusion, we report delays in virologic diagnosis among adults hospitalized for RSV infections. The missed opportunities to recognize the infection early could affect clinical decision making and patient outcomes. Our findings indicate the need to establish efficient, pragmatic diagnostic strategies for RSV in this patient population.

## SUPPLEMENTARY DATA

Supplementary materials are available at *The Journal of Infectious Diseases* online. Consisting of data provided by the authors to benefit the reader, the posted materials are not copyedited and are the sole responsibility of the authors, so questions or comments should be addressed to the corresponding author.

jiz236_suppl_Supplementary_Figures

## Notes

***Acknowledgments.*** We thank David Rivas and Katia Boven for their review comments on the manuscript. We are also grateful to Guy de La Rosa for his input into the study. Medical writing support was provided by Patrick Hoggard from Zoetic Science, Macclesfield, United Kingdom, an Ashfield Company. Support for medical writing assistance was provided by Janssen.

***Financial support.*** This work was supported by Janssen Pharmaceuticals.

***Potential conflicts of interest.*** N. L. has received honoraria for consultancy work from Janssen Pharmaceuticals, Shionogi, and Seqirus. E. E. W. has research contracts from Gilead, Janssen, and Merck Sharp & Dohme and is an unpaid consultant to Novavax and Pfizer. I. S., R. S., and J. Z. are employees of IQVIA, a healthcare consulting firm hired by Janssen Pharmaceuticals. V. W., D. M., and R. F. are employees of Janssen Pharmaceuticals and may be Johnson & Johnson stockholders. All authors have submitted the ICMJE Form for Disclosure of Potential Conflicts of Interest. Conflicts that the editors consider relevant to the content of the manuscript have been disclosed.

Presented in part: European Respiratory Society International Congress, Milan, Italy, 9–13 September 2017; Infectious Diseases Week Conference, San Diego, California, 4–8 October 2017. laishunn@ualberta.ca
