# Supplementary figures and images for: Delayed Diagnosis of Respiratory Syncytial Virus Infections in Hospitalized Adults: Individual Patient Data, Record Review Analysis and Physician Survey in the United States
Source: J Infect Dis. 2019 May 9;220(6):969–79. doi: 10.1093/infdis/jiz236 (PMC6688061; doi:10.1093/infdis/jiz236)

Supplementary Figure 1

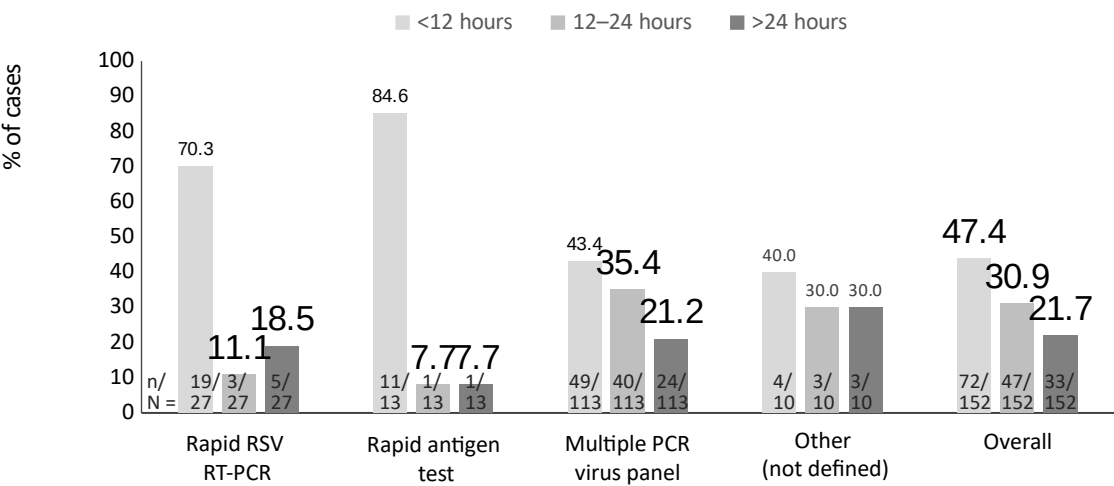

Supplement: jiz236_suppl_Supplementary_Figures [file jiz236_suppl_supplementary_figures.pdf]
